# Biogeographic patterns of biosynthetic potential and specialized metabolites in marine sediments

**DOI:** 10.1038/s41396-023-01410-3

**Published:** 2023-04-15

**Authors:** Alexander B. Chase, Alexander Bogdanov, Alyssa M. Demko, Paul R. Jensen

**Affiliations:** 1 Department of Earth Sciences, Southern Methodist University, Dallas, TX USA; 2 Center for Marine Biotechnology and Biomedicine, Scripps Institution of Oceanography, University of California at San Diego, La Jolla, CA USA; 3 Center for Microbiome Innovation, University of California at San Diego, La Jolla, CA USA

**Keywords:** Biogeography, Metabolomics, Microbial ecology, Environmental chemistry, Metagenomics

## Abstract

While the field of microbial biogeography has largely focused on the contributions of abiotic factors to community patterns, the potential influence of biotic interactions in structuring microbial communities, such as those mediated by the production of specialized metabolites, remains largely unknown. Here, we examined the relationship between microbial community structure and specialized metabolism at local spatial scales in marine sediment samples collected from the Long-Term Ecological Research (LTER) site in Moorea, French Polynesia. By employing a multi-omic approach to characterize the taxonomic, functional, and specialized metabolite composition within sediment communities, we find that biogeographic patterns were driven by local scale processes (e.g., biotic interactions) and largely independent of dispersal limitation. Specifically, we observed high variation in biosynthetic potential (based on Bray-Curtis dissimilarity) between samples, even within 1 m^2^ plots, that reflected uncharacterized chemical space associated with site-specific metabolomes. Ultimately, connecting biosynthetic potential to community metabolomes facilitated the in situ detection of natural products and revealed new insights into the complex metabolic dynamics associated with sediment microbial communities. Our study demonstrates the potential to integrate biosynthetic genes and metabolite production into assessments of microbial community dynamics.

## Introduction

The biogeographic processes that generate and maintain microbial diversity, such as environmental selection, dispersal, and species interactions, offer insights into the mechanisms that structure microbiomes [1]. At large (i.e., continental) spatial scales, community structure is primarily driven by dispersal limitation with environmental filters selecting for specific taxa at increasingly smaller spatial scales [2, 3]. At local spatial scales, there is an assumption that biotic interactions (e.g., predation, competition), including those mediated by chemical communication [4], largely account for the differences among communities (beta-diversity) [5]. The wide array of biologically active microbial specialized metabolites produced in response to both environmental cues and species interactions likely contribute to differences in community composition at small spatial scales [6, 7]. However, understanding how these complex chemical interactions contribute to community dynamics has largely been ignored.

While there is a growing appreciation for microbial chemical ecology [8], challenges associated with the in situ detection of specialized metabolites has hampered our understanding of their effects on community dynamics. For instance, marine sediment bacteria are prolific producers of small molecule natural products or specialized metabolites [9] that include potent antibiotics and cytotoxins. Yet, most of our knowledge of natural product production is inferred from lab cultures, providing limited insights into their ecological roles within a community context [10, 11]. Due to these challenges, molecules mediating such interactions are often inferred from genomic potential as estimated through the detection of natural product biosynthetic gene clusters (BGCs) or other biosynthetic enzymes [12, 13]. These environmental surveys have identified habitat-specific patterns of biosynthetic potential [14, 15], suggesting that specialized metabolites exhibit a biogeographic signal in concordance with taxonomic composition. Although these types of analyses are valuable, the environmental factors that elicit BGC expression remain largely unknown, as well as the concentrations at which compounds occur in nature and their roles in mediating community interactions. Given the recent advancements in mass spectrometry and other analytical tools, the in situ detection of microbial natural products [16, 17] and their influence on community structure remains an essential frontier for the field of microbial ecology.

Here, we sought to focus on both biosynthetic potential and realized natural product production in the context of marine sediment microbial community structure across varying spatial scales. To do so, we compared microbial community composition from 176 marine sediment samples collected from the Long-Term Ecological Research (LTER) site in Moorea, French Polynesia. While benthic cover, nutrient input, and microbial communities associated with coral reefs and the surrounding seawater have been tracked at the Moorea LTER, little work has been done on the sediment microbial communities. Given that shifts in sediment microbiomes can impact above-sediment community health and dynamics [18, 19], we aimed to broaden our understanding of this well-studied ecosystem.

To determine how microbial community structure and specialized metabolism are linked at local spatial scales, we employed a multi-omic approach to characterize the taxonomic composition, functional and biosynthetic potential, and community metabolomes within reef-associated sediments. We constrained our sampling to a single LTER site (LTER2) to minimize negative correlations between taxonomic community similarity and geographic distances (“distance-decay” effects), thereby limiting environmental variation and increasing our ability to determine if community metabolomes influence composition [3]. Furthermore, we employed a partially nested sampling design across eight replicate plots to provide sample comparisons ranging from cm to km scales within this relatively homogeneous environment (Fig. S1A). In doing so, we expected that variation in community composition (beta-diversity) would largely be independent of distance effects (i.e., high community similarity with low slope over the distance-decay curve). Rather, we hypothesized that local site differences could be linked to biosynthetic potential and community metabolomes, as evidenced by low compositional similarity in BGC composition and specialized metabolite production. Although most compounds detected using untargeted environmental metabolomics could not be identified, correlations between compounds and community structure were nonetheless established. Our paired metagenomic-metabolomic approach also facilitated linkages between unknown metabolites and their candidate biosynthetic machinery, which is useful for future natural product discovery efforts.

## Results

### Community patterns in marine sediments

Sediment samples were collected from eight replicate 1 m^2^ plots within and around the Long-Term Ecological site LTER2 in Moorea, French Polynesia (Fig. S1A). LTER2 is characterized by high nitrogen levels (all sites = 0.5 ± 0.06 %N based on algal tissue; Fig. S1B) and steady coral degradation over time (Fig. S1C). The eight collection sites (depth range 0.6–3.1 m) could be further defined as back (*n* = 4) and fringing (*n* = 4) reef, seven of which were characterized by light colored, relatively coarse sediments consistent with high calcium carbonate content. One fringing reef site (site 6) was dominated by algae and characterized by fine dark sediment containing small rocks. Our nested sampling design (16 subsamples × 8 sites + 4 transects per site = 176) allowed for 16 S rRNA gene community composition comparisons across a range of spatial scales. We observed similar community compositions across all sites at broad taxonomic levels with *Proteobacteria* and *Cyanobacteria* dominating the communities (Fig. S2A). At finer-taxonomic levels, as assessed using 16 S rRNA gene amplicon sequence variants (ASVs), we observed a distinct separation between the algae-dominated site (site 6) and the other reef sites (Fig. S2B), thus supporting previous findings that the transition between reef and algae-dominated habitats is reflected in microbial community composition [20].

**Fig. 1:**
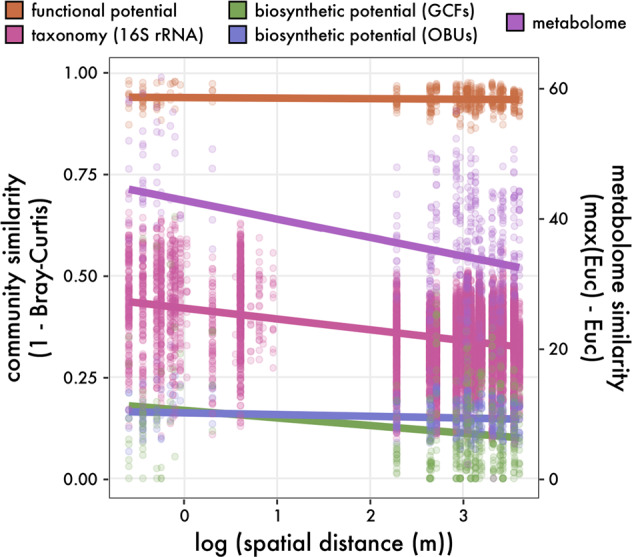
Biogeography of microbial community structure in reef-associated marine sediments. Solid lines denote the least squares linear regression across spatial scales for taxonomic, functional, biosynthetic potential (assessed based on both operational biosynthetic units (OBUs) of ketosynthase/condensation domains and gene cluster families (GCFs)), and community metabolomes. Each point represents a pairwise comparison between community samples with distances derived from Bray-Curtis metrics for all measurements except for metabolomes, which used Euclidean distances.

To assess changes in community composition across cm to km scales within a more homogeneous environment, we excluded site 6 and narrowed our focus to the reef-dominated sediment samples (inset, Fig. S1A). We found that microbial community composition was largely structured at local scales (Fig. S3A; Bray-Curtis, *p* < 0.001 [PERMANOVA]), with site effects accounting for 18.2% of total variation and reef type (fringing vs. back reef) contributing an additional 7.2% (Table 1; *p* < 0.001 [PERMANOVA]). This was further supported by a distance-decay curve demonstrating a significant, negative slope with community similarity decreasing as a function of geographic distance (slope = −0.000013, *p* < 0.001; pink line in Fig. 1). While a small negative slope was observed, distance effects only explained 3.3% of the total variation in community composition (Table 1; reporting *R*^2^ [linear regression]), suggesting that factors other than dispersal limitation are responsible for community differences. Indeed, when we corrected for ASV phylogenetic relatedness (Weighted UniFrac), we observed a significantly shallower slope (Fig. S3C; slope = −0.000003, *p* < 0.001) suggesting that community variation over spatial distances was largely associated with differences among closely related taxa. Given the high correlation between beta-diversity metrics (Spearman’s ρ = 0.77, *p* < 0.001 [Mantel test]), the distance-decay patterns indicate that most compositional variation was due to local scale variation driven by site effects.

**Table 1:** Results from permutational analysis of variance (PERMANOVA) for the effects of site, reef type (back vs. fringing), and geographic distance on microbial community metrics.

	Taxonomic composition			Functional composition	Biosynthetic potential		Realized potential
	ASV (Bray-Curtis)	ASV (UniFrac)	Metagenomes	Pfam	OBUs	GCFs	Metabolome
Site (*R2*)	0.18***	0.17***	0.25**	0.22	0.19***	0.25***	0.28***
Reef type (*R2*)	0.07***	0.07***	0.07**	0.07*	0.04**	0.06**	0.09***
Geographic distance (*R2*)	0.03***	0.01***	<0.0001	<0.01	0.01**	0.01**	0.01***

Percent of variance explained depicted as *R*^2^ values for each effect with level of significance (*p < 0.05, **p < 0.01, ***p < 0.001).

*ASV* amplicon sequence variants of the 16 S rRNA gene, *Pfam* protein families, *OBU* operational biosynthetic unit, *GCF* gene cluster family.

### Functional conservation and biosynthetic variation across sites

Given the negative, shallow distance-decay slopes for taxonomic composition observed across small (<1 m) to large (ca. 1 km) spatial scales (Fig. S3C), we next asked whether functional potential exhibited similar patterns. To do so, we generated shotgun metagenomic data from 36 samples (mean_depth_ = 205 M reads) across all eight sites to initially compare algal to reef samples. Due to possible sequencing biases, we first confirmed that the taxonomic patterns derived from the metagenomic data recapitulated the 16 S rRNA gene results (Table 1; Fig. S3D) and that site differences were consistent across technical replicates (Fig. S3E). Subsequently, we investigated functional potential across communities that may indicate environmental differences within LTER2. In terms of primary metabolic pathways, all sediment communities were similarly enriched in nitrogen cycling and methanogenesis genes (Fig. S4A) and common glycoside hydrolase genes related to the degradation of simple carbon substrates like oligosaccharides and starch (Fig. S4B). To compare functional potential with community composition, we again excluded the algae-dominated site 6 and found that 21.9% of the variation in total functional composition, based on protein family (Pfam) abundances, was explained by local site effects. This relationship did not significantly differ across sites (Table 1; *p* > 0.05 [PERMANOVA]), as we observed high functional redundancy across the reef sediment communities that was independent of distance effects: i.e., geographic distance accounted for only 0.3% of functional variation (orange line in Fig. 1; reporting *R*^2^ [linear regression]: slope = −0.0000011, *p* > 0.05). Similar to taxonomic composition, 7.4% of the total functional variation was significantly associated with reef type (back vs. fringing reef; *p* < 0.05 [PERMANOVA]), possibly owing to higher N input from nearshore storm runoff at the fringing reef sites [21].

Both taxonomic and functional variation analyses indicate that local (within) site processes are contributing to differences among microbial communities. Given that specialized metabolites can mediate biotic interactions [8], we further explored these functional differences across sediment communities by focusing on biosynthetic potential. To reduce biases introduced from assembly-based methods, we first extracted all ketosynthase (KS) and condensation (C) domains from the unassembled data, which are integral to the biosynthesis of polyketides (PKs) and nonribosomal peptides (NRPs), respectively. After clustering domains into operational biosynthetic units (OBUs) at 80% amino acid similarity, based on the prediction that domains in the same OBU will encode the biosynthesis of similar natural products [22, 23], we found high community dissimilarity in OBU composition across sites with a significant, but shallower distance effect compared to taxonomic composition (slope = −0.0000033, *p* < 0.01; blue line in Fig. 1). Distance effects only explained 1.1% of OBU compositional variation across samples while site effects explained 19.3% of variation and reef type (fringing vs. back reef) contributing an additional 4% (Table 1; both *p* < 0.01 [PERMANOVA]). Thus, broad community functions (i.e., primary metabolism) did not differ over geographic distances, but specialized microbial functions (i.e., specialized metabolism) exhibited fine-scale local differences among sediment communities.

**Fig. 2:**
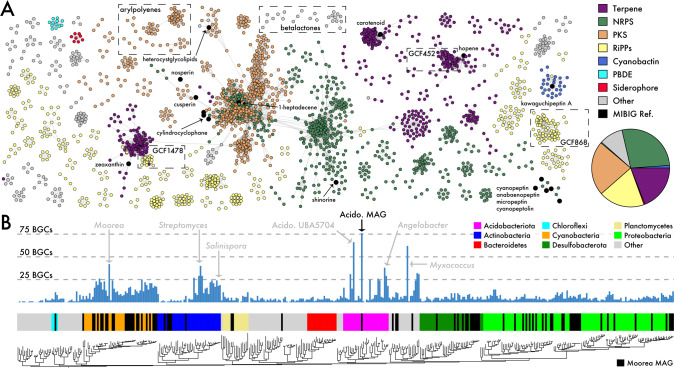
Biosynthetic potential across marine sediment communities. **A** Similarity network of identified biosynthetic gene clusters (BGCs) clustered into gene cluster families (GCFs), colored by BGC classification. Inset pie chart depicts relative proportion of BGC classes. Black nodes indicate known reference BGCs from the MIBIG database. Singleton and doubleton GCFs not shown. **B** BGC distributions across the bacterial phylogeny colored by phyla. Moorea MAGs denoted in black. A subset of reference genomes/MAGs indicated in light gray.

From the assembled metagenomes, we reconstructed 3,137 biosynthetic gene clusters (BGCs) across all samples (Fig. S5B). These were predominantly associated with NRP and terpene biosynthesis. After clustering these BGCs into 1147 gene cluster families (GCFs; Fig. 2A), we found that only 14 were related to experimentally characterized BGCs [24], the products of which include peptins, carotenoids, and heterocyst glycolipids (Fig. S5A). The remaining GCFs represent uncharacterized biosynthetic potential. We observed minimal GCF overlap among samples with only two shared across all eight sites. Both could be assigned to *alphaproteobacterium* based on metagenome-assembled genome (MAG) classifications (order: *Kiloniellales*; abundance_16S_ = 0.38 ± 0.45%). These ubiquitous GCFs were predicted to encode an unknown thioaminidated ribosomal peptide (TfuA-related BGC) and a hopene-like terpene (GCF868 and GCF452 in Fig. 2A, respectively). Only one GCF was found in all reef-associated sites but absent in the algae-dominated samples (site 6). This GCF encoded a hybrid terpene-bacteriocin, with some similarity to the BGC associated with the common carotenoid zeaxanthin (Fig. 2A), and was observed in a group of *Desulfobacterota* MAGs (order: *Desulfobacterales*; abundance_16S_ = 5.95 ± 2.6%). Most of the GCFs (accounting for 679 of the 3137 BGCs) shared no similarity with other GCFs within or across sites and thus were represented as unconnected nodes in the network. This lack of GCF overlap resulted in low compositional similarity with high GCF compositional differences across geographic distances (Table 1; slope = −0.0000097, *p* < 0.01; green line in Fig. 1), thus indicating unique biosynthetic gene assemblages within each sample.

To further explore primary and specialized taxonomic distributions, we examined the metabolic and biosynthetic potential of the 789 MAGs (Table S1), which accounted for 84% (2635 of 3137) of all assembled BGCs. These MAGs were predominantly identified as *Cyanobacteria* and *Proteobacteria*, mirroring our community composition results (Fig. S2A). We first analyzed our medium- (*n* = 80; completeness > 70%, contamination < 10%) and high-quality (*n* = 88; completeness > 85%, contamination < 10%) MAGs (dereplicated to *n* = 104) for primary metabolic potential. In terms of the dominant primary metabolic pathways across communities (Fig. S4A), *Gammaproteobacteria* MAGs encoded genes for the (de)nitrification and N fixation pathways while *Desulfobacterota* MAGs converted methylamine to ammonia as part of methanogenesis. In terms of biosynthetic potential, we compared our dereplicated medium- and high-quality MAGs to a variety of closely related genomes/MAGs from taxa that are known for high abundances of BGCs and specialized metabolite production (Fig. 2B). In particular, we assembled a high-quality *Acidobacteriota* MAG135.14 (91% complete, 4% contamination, genome size = 11.6 Mbp) from a single sample that encoded 76 BGCs (2.2 Mbp or 18.7% of the genome, average BGC length = 28.6 kbp), a number that surpasses any known MAG or genome including those documented across a global metagenomic dataset [25]. While the fragmentation of many BGCs likely skews BGC content, these approaches offer insights into unexplored chemical space and taxa. Together, both OBU and GCF metrics demonstrate high levels of variability in the biosynthetic potential of sediment microbial communities, even within 1 m^2^ sites. This likely reflects a wealth of uncharacterized biosynthetic potential that was not previously documented in global databases.

### Connecting biosynthetic potential to community metabolomes

**Fig. 3:**
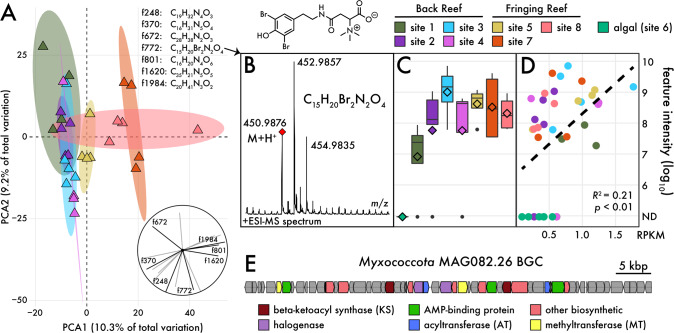
Community metabolomes and candidate producers. **A** Principal Component Analysis (PCA) showing differences in community metabolomes. Colored ellipses represent 75% confidence intervals around each site. Inset depicts contributions of the top molecular features to the principal dimensions with black lines denoting features with predicted molecular formulas shown above. **B** MS2 fragmentation of feature772 with diagnostic bromination signature, calculated mass, and probable molecular structure. **C** Abundance of feature772 across sites based on feature intensity in MS1 chromatograms. Black bars represent medians, diamonds represent means. **D** Linear regression of feature772 with the abundance of *Myxococcota* MAG082.26 (assessed by reads per kilobase mapped, RPKM). **E** Candidate BGC for feature772. Arrows represent individual genes and colored by predicted function.

While gene-based surveys are indicative of biosynthetic potential, many BGCs are dependent on specific environmental triggers for expression [26] and thus remain silent under laboratory conditions [27]. Therefore, to investigate how biosynthetic potential translated to compound production, we utilized untargeted liquid chromatography–high-resolution mass spectrometry (LC-HRMS) to analyze extracts from the sediment samples (*n* = 36) used for the metagenomic analyses. In total, we identified 2164 unique molecular features and show that the major taxonomic differences between the reef and algae-dominated communities (Fig. S2B) translated to the metabolomes (Fig. S6A). After removing controls and features from the algae-dominated site 6, the reef-associated sediment metabolomes displayed clear site-specific signatures (Fig. 3A; Euclidean distance). Indeed, the community metabolome strongly reflected local site and reef type (fringing vs. back reef), accounting for 27.5% and 9.4% of the total variation, respectively (Table 1; both *p* < 0.001 [PERMANOVA]). The site-specific metabolome signatures were strongly reflected in our distance-decay curves (purple line in Fig. 1) where metabolite similarity was greatest within sites.

While local site effects accounted for an increasing percent of variation explained when moving from taxonomy (16 S rRNA gene) to biosynthetic potential (GCF) to metabolomes (Table 1), we did not observe strong correlations across these metrics. For instance, metabolite composition did not correlate with either 16 S rRNA gene ASV (Spearman’s ρ = −0.02, *p* > 0.05 [Mantel test]) or OBU compositions (Spearman’s ρ = 0.16, *p* > 0.05 [Mantel test]). Rather, these compositional metrics may indicate that finer resolution is needed to correlate taxa, biosynthetic genes, and compounds. When we assessed the correlation between metabolomes and fine-scale taxonomic marker genes (i.e., diversity below the 16 S rRNA gene) derived from metagenomic data [28], we found higher congruence between metabolomes and taxa (Spearman’s ρ = 0.37, *p* < 0.01 [Mantel test]). These results suggest that finer levels of taxonomic resolution are needed to resolve relationships between specialized metabolites and their producers. Finally, these results indicate that deep metagenomic sequencing, as performed here, can promote connections between the inherently complex multi-omic datasets generated from environmental samples.

To determine how microbial community metabolomes are structured at local scales, we applied machine learning approaches to identify molecular features driving metabolomic variation across samples. Cheminformatic tools associated with the GNPS online platform facilitated the analysis of our large LC-MS datasets and the identification of molecules based on the comparison of MS/MS fragmentation spectra with over 580,000 library spectra [29]. We visualized the chemical space present in the sediment metabolomes using feature-based molecular networking [30] (Fig. S6B). Comparable with the biosynthetic potential encoded in the metagenomes, we observed a large proportion of singleton nodes in the network (1770, 81.8%) indicating the detection of large numbers of structurally unrelated compounds. In addition, 357 features (16.5% of the total) were observed at one site and 185 (8.5%) were shared between two sites, further highlighting the fine-scale spatial differences in community metabolomes. Further multivariate and random forest analyses revealed unidentified molecular features that were responsible for sample and site differences (inset in Fig. 3A).

While a majority of the compounds associated with the metabolomic differences among sites could not be identified, even after manual inspection, database searches [31], and other cheminformatic predictions [32], we were nonetheless successful in the identification of several natural products in the marine sediment extracts. The initial structural predictions based on MS/MS spectra comparisons with GNPS suggested 388 known features (Fig. S6B); however, many of these annotations proved spurious after manual inspection of their MS/MS mirror plots to reference spectra. Among the confidently identified features, most could be described as lipids, chlorophyll breakdown products (e.g., pheophorbide), and carotenoids (e.g., fucoxanthin). We also confidently identified several microbial specialized metabolites including coumarin derivatives and the cyanobacterial derived cyclic depsipeptides hantupeptins A-C [33, 34], which were only detected at site 8 (Fig. S6B). The latter of these features were initially annotated by GNPS as the closely related antanapeptins A-C [35], but manual inspection of the MS/MS spectra revealed peaks corresponding to *m/z* = 228.1033 and 246.1112 (Fig. S6C), which are diagnostic for the proline-3-phenyllactic acid fragments in the hantupeptins [33]. While the BGC for hantupeptins is unknown, we identified a highly fragmented NRPS BGC from the site 8 metagenome with homology to the distantly related anabaenopeptin BGC (Fig. S6D). This partial BGC included an LCL condensation domain and an adenylation domain selecting for valine (Fig. S6E), both of which are predicted for hantupeptin biosynthesis [33, 34]. Nevertheless, most molecular features could not be annotated and represent unexplored chemical space associated with sediment metabolomes.

### Linking metabolites to producers

Pairing metagenomic and metabolomic datasets provides opportunities to link compounds to their cognate BGCs based on correlations in relative abundances and predicted biochemistry. While most molecular features provided only minimal structural information, we identified one with the diagnostic MS isotopic pattern of a dibrominated compound with the most intensive peak *m/z* = 452.9857 [M+H]^+^ and the lowest *m/z* = 450.9876 [M+H]^+^ (Fig. 3B). The calculated molecular formula (C_15_H_20_Br_2_N_2_O_4_) had no matches in the MarinLit database [31] and likely represents a new natural product. Extracts containing the highest amounts of this compound were pooled (Fig. S7A) and fractionated for LC-HRMS (Fig. S7B). The spectra suggested an alkaloid (Fig. S7C) consisting of a decarboxylated dibromotyrosine (high intensity peak *m/z* = 291.8975 [36], Fig. S7D) fused over an amide bond to an aspartic acid derived betaine, which corresponds to the observed mass loss of 159.089 Da and the high polarity (water soluble) of the compound (Fig. S7B). This new compound is structurally related to sponge-derived alkaloids [37]. However, due to the low compound yield, we were unable to explore its molecular structure further using nuclear magnetic resonance (NMR). This compound was observed at all sites except the algae-dominated site 6 with its highest abundance at site 3 (Fig. 3C). After correlating MAG abundances with the intensity of this dibrominated compound, we identified the *Myxococcota* MAG082.26 as a possible producing organism and a group of *Desulfobacterota* MAGs that have the potential to use this dibrominated molecule as an oxidant during respiration.

The high-quality *Myxococcota* MAG082.26 (93% complete) has a genome size of 7.7 Mbp and is distantly related to all reported genome or MAG sequences in this phylum, with its closest relative (77.4% average nucleotide identity [ANI]) belonging to the MAG-associated UBA9160 family. MAG082.26 was assembled from site 3, which contained the highest concentrations of the dibrominated aromatic metabolite, and was positively correlated with the mass of this compound across all samples (Fig. 3D; *R*^2^ = 0.21 [linear regression]). The biosynthesis of this molecule requires the halogenation of the aromatic phenyl ring (i.e., dibromotyrosine), which has been previously shown in marine myxobacterial metabolites such as salimabromide [38]. *Myxococcota* MAG082.26 harbors nine BGCs of which one contains two tetracycline 7-halogenase genes that share homology to brominases involved in the biosynthesis of the 2-arrylpyrrole moiety in pentabromopseudilins (MIBIG: BGC0000890/1) and bromopyrroles/bromophenols (MIBIG: BGC0001465). While the gene architecture of this BGC is unique (Table S2), it lacks the NRPS component (i.e., adenylation domain) expected for the biosynthesis of compound *m/z* = 452.9857 (Fig. 3E). While this remains the best candidate BGC assembled from our metagenomic data, more work is needed to confirm this connection.

While investigating linkages between MAGs, BGCs, and the dibrominated metabolite, we identified a group of five related MAGs (ANI ranges 77.2–98.5%) belonging to two bacterial families within the order *Desulfobacterales* that were highly correlated with the presence of this compound (*R*^2^ = 0.46 ± 0.01 [linear regression]). While these *Desulfobacterota* MAGs lacked the biosynthetic machinery to produce compound *m/z* = 452.9857, they encode a reductive dehalogenase (*rdh*) operon (Fig. S8A/B) along with genes for cobalamin biosynthesis, a key cofactor associated with reductive dehalogenation [39]. Moreover, the *rdh* operon is co-localized with several genes encoding electron transport proteins (i.e., ferredoxins and NADH-quinone reductase complexes; Fig. S8A), providing evidence that these microorganisms have the potential to use dibromophenol as a terminal electron acceptor during respiration. These findings add to the complex functional roles of natural products and support growing evidence that community metabolomes are shaped both by compound production and subsequent modification by other members of the community [26, 40].

## Discussion

While the field of microbial biogeography has largely focused on the role of abiotic variables in structuring microbial distributions, there is growing appreciation for the role of microbial natural products in mediating biotic interactions within microbiomes [8]. The ability to assess environmental metabolomes is rapidly improving [16, 41], providing opportunities to transcend culture-based and synthetic community approaches or biosynthetic inferences derived from co-occurrence networks [42]. Here, we assessed the relationships between community metabolomes and composition at varying spatial scales by minimizing environmental variation through extensive sampling within a relatively homogeneous environment. We observed distinct biogeographic patterns associated with taxonomic composition, functional genes, biosynthetic potential, and the environmental detection of natural products. Finally, our multi-omics approach facilitated potential connections between metabolites, producing organisms, and those that may benefit from their production.

By reducing environmental variation through sampling within a single LTER site, we found evidence for biogeographic patterns that were largely independent of dispersal limitation. For example, geographic distance did not explain a large portion of community variance across all community measurements (<4% of total variance explained; Table 1), indicating that local, within site processes (e.g., biotic mechanisms) are driving community differences. This was evidenced from broad inter-habitat differences between the reef- and algae-dominated sites (Fig. S2C) and the high community similarity across spatial distances (Fig. 1), a pattern that was accentuated after correcting for phylogenetic relatedness (Fig. S3C). While ecological drift can account for local scale differences [43], our results suggest that biotic interactions, as measured by biosynthetic potential and specialized metabolite production, are linked to local biogeographic patterns. In both cases, we observed high community dissimilarity in biosynthetic potential (Fig. 1) and pronounced local site effects in community metabolome composition (Fig. 3A; Table 1). These patterns are largely congruent with observations of soil fungal communities, where local scale patterns were driven by extracellular enzyme production [44] that was not reflected in community composition.

Given the increasing awareness that natural products play important roles in structuring microbial communities, connecting chemical compounds with their producers (and associated biosynthetic machinery) and ultimately to ecological interactions remains a priority [45]. Despite major methodological advances, <5% of metabolites detected in marine systems can be identified [46] and fewer could be linked to ecological functions. Our results further demonstrate the limitations of cheminformatic tools for the identification of environmental specialized metabolites and the importance of stringent manual curation. In addition, untargeted metabolomics may miss low concentration compounds produced by members of the “rare biosphere” that are important contributors to the specialized metabolite pools in marine sediments [47]. Finally, correlation analyses built from co-occurrence networks of taxonomy and metabolites typically rely on coarse taxonomic markers, such as the 16 S rRNA gene. Given the lack of concordance between these parameters in our results, combined with evidence of high trait variation within 16S-defined taxa [48, 49], it appears that more highly resolved taxonomic marker are needed to assess relationships between metabolomes and the structure of complex microbial communities at fine spatial scales. A likely contributor to this observation is the fine-scale phylogenetic conservation in specialized metabolite production and BGC composition within taxa [50–53]. Nonetheless, at large spatial scales and across disparate environments, broad patterns of biosynthetic potential [14, 15, 54, 55] and the abundance of microbially-related metabolites have been shown to vary in a habitat-specific manner [56].

The integration of environmental metabolomes into microbiome research provides a new framework to address questions in microbial chemical ecology. The complex chemical landscapes associated with sediment microbial communities (Fig. 3A) coupled with the unprecedented biosynthetic potential of sediment MAGs (Fig. 2B) supports recent evidence that poorly studied phyla such as *Acidobacteriota* [57] and *Myxococcota* hold promise for future natural product discovery efforts. While this approach can facilitate the prioritization of BGCs for heterologous expression based on the predicted novelty of their biosynthetic products (Fig. 2A), it remains difficult to connect molecules with their cognate BGCs when dealing with complex microbial communities [58], as we have attempted to do with the hantupeptins (Fig. S6). Furthermore, the dynamics of specialized metabolism are complex, with molecules not only produced but also degraded or metabolized. Our detection of a dibrominated tyrosine metabolite (Fig. 3B) and a MAG containing the genetic potential to metabolize this compound provide examples of the interplay between these processes. Ultimately, a major goal remains to better understand the roles of environmental metabolomes in shaping microbial community composition.

## Conclusion

Recent methodological advancements have enabled the incorporation of metabolomics into assessments of microbial diversity. Multi-omic studies of this type can begin to reveal the potential roles of specialized metabolites in structuring microbial communities in complex environmental biomes. These molecules likely mediate a myriad of largely undefined interactions among community members and the environment. A better understanding of environmental metabolomes and their functional roles will provide a new dimension to assessments of microbial diversity and distributions.

## Methods and materials

### Field site and sample collection

Sediment samples were collected from the barrier reef lagoon surrounding the island of Moorea, French Polynesia. We collected samples adjacent to the back and fringing reefs within eight replicate 1 m^2^ plots in and around the Long-Term Ecological Research (LTER) Site 2 (Fig. S1A). Plot locations were selected based on proximity to either the fringing reef (*n* = 4) or the back reef (*n* = 4). Sites 1–4 were designated as back reef sites with a depth range of 1.83–3.05 m. Site 5–8 were initially designated as fringing reef sites (<150 m from shore) that ranged in depth from 0.61–1.83 m, while site 6 was further designated as algae-dominated on the predominance of seaweeds and lack of living coral.

Within each 1 m^2^ plot, 16 subsamples were taken using a gridded design (Fig. S1A; *n* = 128). Additionally, sediments were sampled every meter along a 4 m transect in each cardinal direction away from the 1m^2^ plots (*n* = 256, 128 from plots, 128 from transects). All sediments samples (~80 g each) were collected in sterile Whirl-Pak bags and transferred on ice back to the field station for processing. Approximately 7 g of each sediment sample was transferred to a 15 mL Falcon tube with 7 mL of RNALater for subsequent community analyses. All tubes and the remaining sediment were stored at −20 °C until processing except when in transit to Scripps Institution of Oceanography when dry ice was used.

LTER data related to N content, heat stress, and coral degradation were sourced from publicly available data (see [21, 59]). Briefly, in R, public geospatial data from LTER monitoring was interpolated by converting geographic coordinates for our collection sites to geospatial vectors (st_as_sf in ‘sf’ package) and applying a kriging algorithm (kriging in ‘kriging’ package) to predict environmental values at our sites.

### 16S rRNA gene sequencing and analysis

All quadrat samples (*n* = 128) and transect tips (*n* = 32) were processed for DNA extraction. Mid-point transect samples (*n* = 4) were also processed for site 3, which was qualitatively identified as having the highest coral cover. All remaining transect samples from site 6 were also processed to increase spatial comparisons among the algae-dominated site (*n* = 12). In total, 176 samples were processed for 16 S rRNA gene sequencing. For DNA extraction, ~2 g of sediment in RNALater was processed by the Center for Microbiome Innovation (CMI) at UC San Diego using the MagAttract PowerSoil DNA KF kit (Qiagen). Samples were subsequently sequenced with the MiSeq System PE150 platform (Illumina) using the V4 region 515 forward primer (GTGYCAGCMGCCGCGGTAA) and the updated 806 reverse primer (GGACTACNVGGGTWTCTAAT) recommended by the Earth Microbiome Project [60]. For increased sequencing depth to capture the rare biosphere, two separate sequencing runs were performed for community analyses (see Supplemental Materials).

### Metagenomic sequencing and analysis

For a subset of samples (*n* = 36) representing all eight sites, shotgun metagenomic libraries were constructed using a Nextera XT DNA library preparation kit (Illumina) at the CMI. To reduce sequencing biases, 20 of 36 samples were sequenced in triplicate on a HiSeq system (Illumina) with 150-bp paired end reads. Technical replicates were initially processed as independent samples and assessed for variance in taxonomic composition (see below). Upon validation, technical replicates were combined for all downstream analyses. The remaining samples were sequenced on a NovaSeq 6000 System (Illumina) with 150-bp paired end reads to achieve similar sequencing depth (mean_depth_ = 205 M reads). Raw reads were quality trimmed with adapters removed using the BBMap toolkit (bbduk.sh) [61]. Processed reads were then analyzed using both a read-based (for taxonomic, functional, and KS/C biosynthetic potential) and assembly-based approaches (see Supplemental Materials).

### Sediment metabolomics and mass spectrometry

For the same samples used to construct shotgun metagenomes, we partitioned sediments (wet weight 50 g) for chemical extractions with 100 mL 1:1 methanol dichloromethane mixture. The solvent was filtered and evaporated under reduced pressure. The extracts were resuspended in HPLC grade methanol with the supernatant removed and diluted to 1 mg/mL for LC-HRMS analysis. Process controls were prepared analogously using the same glassware. LC-HRMS analysis was performed on a 6530 Accurate-Mass QToF with ESI-source (Agilent) coupled with a 1260 Infinity HPLC (Agilent) equipped with a 150×4.6 mm Kinetex C18 5 µm column (Phenomenex, USA). HPLC was run starting at 20:80 acetonitrile: water with 0.1 % formic acid (FA) for 2 min followed by an 18 min gradient until 95 % acetonitrile (0.1 % FA), which we held for 2 min and increased to 100 % acetonitrile over 1 min and held for 2 min. We injected 5 µL of a 1 mg/mL sample solution (MeOH) at a flow of 1 mL/min. MS data were acquired over a range 135–1700 *m/z* in positive mode. All solvents were LCMS grade. Paired metagenomic and metabolomic datasets for each sample are available on the Paired Omics Data Platform [62]. Chemoinformatic analyses are detailed in Supplementary Materials.

## Supplementary information

Supplemental Materials and Methods

Figure S1

Figure S2

Figure S3

Figure S4

Figure S5

Figure S6

Figure S7

Figure S8

Table S1

Table S2

## Data Availability

Paired-end shotgun metagenomic and 16 S rRNA amplicon data were deposited in the NCBI Sequence Read Archive under the BioProject PRJNA611818. Public datasets for all metabolomic spectra files are available at massive.ucsd.edu (MSV000091150). All other data, including genomes, and relevant code used can be found at https://github.com/alex-b-chase/mooreaMS.
