# Supplementary material for: Biogeographic patterns of biosynthetic potential and specialized metabolites in marine sediments
Source: ISME J. 2023 Apr 15;17(7):976–83. doi: 10.1038/s41396-023-01410-3 (PMC10284892; doi:10.1038/s41396-023-01410-3)
Supplement: Supplementary file 1 — Supplemental Materials and Methods [file 41396_2023_1410_MOESM1_ESM.docx]

Supplementary Materials for

**Biogeographic patterns of biosynthetic potential and specialized metabolites in marine sediments**

Alexander B. Chase*, Alexander Bogdanov, Alyssa Demko, Paul R. Jensen

*Corresponding author, email: abchase@smu.edu

This PDF file includes:

Extended Materials and Methods

Supplementary Figures and Tables

References

**Extended Materials and Methods**

16S rRNA gene sequencing and analysis (extended)

Raw reads were demultiplexed and combined from the two independent sequencing runs using the BBMap toolkit [1]. Paired-end files were quality trimmed with adapters removed, joined, and processed using the QIIME2 toolkit (v2021.8.0) [2] with operational taxonomic units (OTUs) called using amplicon sequence variants (ASVs) with DADA2 [3]. Taxonomic classifications were assigned using a weighted naïve Bayes taxonomic classifier trained on the 515F/806R region [4] from the SILVA 138.1 reference database [5], which resulted in the removal of ASVs classifying as chloroplast, mitochondrial, and other eukaryotic sequences. From the OTU matrix, we rarefied the composition table by randomly subsampling to a range of quantiles to compute sampling depth as a function of OTU richness. Based on OTU saturation, we rarefied to 1% (n=27,757 reads) from each sample for 100 iterations. To weigh rarer taxa more heavily, we performed a Wisconsin double standardization and rounded each to the nearest integer. Using the rarefied composition table, we calculated a median Bray-Curtis distance matrix to produce a non-metric dimensional scaling (NMDS) plot using the ‘vegan’ package in R (v4.0.3). All samples from site 6 (algal-dominated outgroup site) were removed and analyses were re-run for all downstream statistical analyses. Finally, to test for differences among sites and to estimate the variance explained by reef type (i.e., fringe v. back reef), we performed a permutational multivariate analysis of variance (PERMANOVA) for 999 permutations under a reduced model.

Metagenomic sequencing and analysis (extended)

For the read-based approach, merged paired-end reads and the remaining unmerged forward reads were analyzed for coding regions using prodigal (with the metagenomic flag) [6] for taxonomic, functional, and biosynthetic analyses. Taxonomic compositions were generated using a custom reference database of curated single-copy marker genes [7], as previously described [8]. Similarly, biosynthetic genes were extracted by searching the metagenomic reads against the NaPDoS2 [9] database for ketosynthase (KS) and condensation (C) domains. For both taxonomic and biosynthetic classifications, a preliminary protein search using BLAT [10] extracted preliminary hits to either the taxonomic markers or KS/C domains. Next, we employed a secondary filter using Hidden Markov Models (HMMs) [11] and aligned all filtered reads to each respective database with Clustal Omega (v1.2.0) [12]. Passed reads were placed onto reference phylogenies using pplacer (v1.1.alpha17) [13] for assignment to either taxonomic or biosynthetic classifications. Taxonomic relative abundances were calculated by generating single-branch abundance matrices and normalizing to the total number of genes/domains in each database. The classified KS/C domains were clustered into operational biosynthetic units (OBUs) using cd-hit [14] at 80% similarity [15] and rarefied at the 1% quartile (n=17,206 reads) from each sample for 100 iterations. Functional annotations were assigned using HMMs and filtered based on the gathering threshold (--cut_ga) designated in the PfamA database [16]. Pfam annotations were collated by sample and rarefied to 8,577,932 reads from each sample for 100 iterations. To test the effects of site and coral reef type (back and fringe reef), we generated a Bray-Curtis dissimilarity matrix for taxonomic, functional, and biosynthetic compositions as input for a PERMANOVA for 999 permutations, which were visualized in NMDS plots.

For the assembly-based approach, quality filtered reads were normalized prior to assembly with BBNorm (target=40 mindepth=5). Normalized reads for each sample were independently assembled using the IDBA-UD assembler [17] using the pre-correction flag (mink=30 maxk=200 step=10). After removing smaller contigs (<5000 bp), we predicted coding regions using prodigal and biosynthetic gene clusters (BGCs) using antiSMASH (v5.1.2) [18]. Metagenomic BGCs were compared to reference clusters in MIBIG v2.1 [19] and dereplicated into gene cluster families (GCFs) using BiG-SCAPE (v1.1.2) [20]. The GCFs were clustered based on protein domain sequence similarity and shared domains (distance <0.6). Pairwise distances (squared similarity scores ranging from 0-1) based on the BiG-SCAPE output were visualized in a network with Cytoscape [21]. For GCF composition, we generated a Bray-Curtis distance matrix to construct NMDS ordination plots and perform a PERMANOVA for 999 permutations. Comparisons of BGCs and other operons were visualized using clinker [22].

Assembled contigs from each sample were individually binned into metagenomic assembled genomes (MAGs) by first calculating contig coverage across all samples with bowtie2 (--sensitive --maxins 800) [23]. Using differential coverages and other genomic signatures, we performed an unsupervised binning approach with Metabat2 [24] and assessed the quality and completeness of all binned MAGs using checkM [25]. MAG abundances were further defined across samples by calculating the reads per kilobase of genome size per million reads mapped (RPKM). All MAGs were dereplicated with dRep [26] and classified against the Genome Taxonomy Database [27] with GTDB-Tk [28]. Dereplicated MAGs were assessed for primary metabolic pathways using DRAM [29] under default parameters. Targeted assessment for reductive dehalogenases (*rdhA*) and other queried enzymes were searched with BLAT against characterized *rdhA* enzymes, as previously described [30].

To compare the number of BGCs per MAG, we downloaded all closely related genomes identified by the GTDB classification (Table S1) and other publicly available genomes and MAGs (N=329), including those enriched in biosynthetic potential [31, 32]. First, we reconstructed a multi-locus phylogeny using five single-copy ribosomal marker genes with our high- (completeness >85%, contamination <10%) and medium-quality (completeness >70%, contamination <10%) MAGs (n=183 dereplicated to 101). Single-copy genes were concatenated using Clustal Omega (v1.2.0) to infer a core phylogeny with RAxML v8.2.12 [33] under the PROTGAMMABLOSUM62 model for 100 replicates. All genomes and MAGs in the phylogeny were re-run with prodigal and antiSMASH to standardize gene calling and BGC predictions. Visualization of all phylogenies were generated with iTOL [34].

Sediment metabolomics and mass spectrometry (extended)

A molecular network was created with the Feature-Based Molecular Networking (FBMN) workflow on GNPS (https://gnps.ucsd.edu). The mass spectrometry data were first processed with MZMINE2 [35] and the results were exported to GNPS for FBMN analysis. MS/MS spectra were window filtered by choosing only the top 6 fragment ions in the +/- 50 Da window throughout the spectrum. The precursor ion mass tolerance was set to 1.0 Da and the MS/MS fragment ion tolerance to 0.5 Da. A molecular network was then created where edges were filtered to have a cosine score above 0.7 and more than 4 matched peaks. Further, edges between two nodes were kept in the network only if each of the nodes corresponded to the top 10 most similar nodes. Finally, the maximum size of a molecular family was set to 100, and the lowest scoring edges were removed from molecular families until the molecular family size was below this threshold. The analogue search mode was used by searching against MS/MS spectra with a maximum difference of 500.0 in the precursor ion value. The library spectra were filtered in the same manner as the input data. All matches kept between network spectra and library spectra were required to have a score above 0.7 and at least 4 matched peaks. The molecular networks were visualized using Cytoscape v3.9 [21]. All library spectra matches were manually re-inspected and evaluated.

A preliminary Principal Components Analysis ordination plot was generated for 2164 molecular features found in the 44 samples (Figure S6A; 30 coral, 6 algal, 4 process controls, 2 filter controls, 2 methanol controls). After manually removing all features found in control samples, the remaining 1793 features were normalized using a log_2_ transformation with pareto scaling to generate a Euclidean distance matrix. Next, we performed a permutational multivariate analysis of variance (PERMANOVA) on the reef-associated samples with reef site and type (i.e., fringe v. back reef) as fixed effects for 999 permutations under a reduced model. The Euclidean matrix was used to generate a final Principal Components Analysis ordination plot and to determine which molecular features were driving site differences. Across all features, we noted an apparent break in feature contributions (red line in Figure S6D) and performed additional validation among the top 223 features. Specifically, we cross-referenced the contribution of each feature with random forest analyses conducted with MetaboAnalyst [36] to determine a measurement of permutation importance for each predictor feature. Finally, we manually curated the fragmentation patterns of the predictor features to remove contaminants (e.g., PEG), dereplicate feature adducts (e.g., combine [M+H]^+^ and [M+Na]^+^), and identify diagnostic MS2 fragmentation patterns (e.g., bromination) for the 16 confidently identified features driving separation in our PCA. Based on *m/z* and retention time of features, we predicted putative molecular formulas when possible.

**SUPPLEMENTARY FIGURE LEGENDS**

**FIGURE S1.** Environmental measurements collated from Moorea, French Polynesia. **A)** Satellite view of Moorea with LTER stations. Coordinates of sampling locations for each site around LTER2. Inset shows plot for fine spatial sampling. **B)** Measured nitrogen content and cumulative heat stress on coral reefs in Moorea. Reproduced from Donovan et al. 2020. **C)** Percent of coral cover across the Long-Term Ecological Research (LTER) stations over time. Orange line represents the carbon to nitrogen ratio. Red line indicates sampling year and station of current study. Redrawn and updated from Adam et al. 2021.

**FIGURE S2**. Community composition of sediments around LTER2 station. **A)** Microbial community composition at the phylum level averaged across sites. **B)** Nonmetric multidimensional scaling (NMDS) plot depicting community composition (Bray-Curtis based on 16S rRNA) for each sample (N=176). Circles represent sample colored by site.

**FIGURE S3.** Comparison of community composition patterns based on methodology and distance score of reef-associated sediments. NMDS plot depicting community composition compiled from 16S rRNA data with **A)** Bray-Curtis dissimilarity and **B)** Weighted UniFrac to adjust for phylogenetic similarity. **C)** Distance decay of community compositions in A and B based on distance metric as a function of geographic scale. Note Bray-Curtis dissimilarity is also shown in Fig 1. **D)** NMDS of community composition derived from metagenomic data (N=36). **E)** NMDS of community composition for technical replicates for a subset of metagenomes (n=60; 20 samples x 3 technical replicates). Standard deviations from three technical replicates for each sample (denoted as MXXX) are shown as black lines.

**FIGURE S4.** Functional composition across sediment metagenomes (N=36). Heatmaps show the enrichment of **A)** primary metabolic genes associated with an annotated pathway and **B)** carbohydrate active enzymes involved in the degradation of organic matter labeled by predicted substrate. Hierarchical clustering generated from a Euclidean distance matrix.

**FIGURE S5.** Distribution of biosynthetic gene clusters (BGCs) by site. **A)** Same network as in Figure 2 with nodes colored by site. Singleton nodes not shown. **B)** Average number of BGCs assembled and characterized per metagenome by site.

**FIGURE S6.** Community metabolomes. **A)** Principal Components Analysis (PCA) showing differences in community metabolomes across samples. Inset depicts molecular features contributing to total variation in PCA from Figure 3A, displayed as a rank-order plot. Due to discontinuity delineating significant features (dashed red line), only features to the right of the red line were manually inspected. **B)** Feature-based molecular network generated from sediment extracts with GNPS annotations after manual review. Nodes represent parent ions and are connected by edges based on similarity in MS/MS fragmentation spectra. Singleton nodes not shown. **C)** Support for the detection of hantupeptin A based on calculated molecular formula and analysis of MS/MS fragmentation in sample MO_234. **D)** Candidate biosynthetic gene cluster (fragmented BGC of only two genes) for hantupeptins assembled from sample MO_234. The BGC shares homology to those reported to produce the related anabaenopeptin molecules (numbers denote amino acid similarity). **E)** Only one module recovered in the nonribosomal peptide synthetase (NRPS) gene with a condensation (C) domain catalyzing a LCL reaction, adenylation (A) domain selecting for valine (val), carrier protein (PCP), and thioesterase (TE).

**FIGURE S7.** Mass spectrometry and characterization of feature772. **A)** Base peak chromatogram (BPC) from sample MO_071 that had the highest intensity of feature772. **B)** Extracted ion chromatogram (EIC) for feature772 (*m/z*=452.9857). **C)** HR-MS spectrum of feature772 displaying a group of isotopic peaks diagnostic for a di-brominated molecule. **D)** Suggested structure of feature772 based on calculated molecular formula and analysis of MS/MS fragmentation.

**FIGURE S8.** Reductive dehalogenase (*rdh*) metabolism in *Desulfobacterota* MAGs. **A)** Phylogeny of putative *rdhA* genes from *Desulfobacterota* MAGs (in red) compared to known *rdhA* enzymes (UniProt IDs denoted in tree labels). Branch lengths measured in number of amino acid substitutions per position. Blue triangles indicate nodes with bootstrap support >50%. **B)** Putative *rdh* operons from MAGs compared to a known organohalide respiring bacterium, Desulfoluna spongiiphila strain AA1. *Rdh* operon defined by three essential genes, *rdhA* (reductive dehalogenase enzyme), *rdhB* (membrane-anchoring protein), *rdhC*. Desulfobacterota MAGs also contain additional electron transport complexes, such as ferredoxins and NADH-quinone reductases (*nqr*).

**TABLE S1.** Genome information for binned MAGs.

**TABLE S2.** BLAST and functional annotation of candidate BGC in *Myxococcota* MAG082.26.

1. Bushnell B. BBMap: a fast, accurate, splice-aware aligner. 2014. Lawrence Berkeley National Lab.(LBNL), Berkeley, CA (United States).

2. Bolyen E, Rideout JR, Dillon MR, Bokulich NA, Abnet CC, Al-Ghalith GA, et al. Reproducible, interactive, scalable and extensible microbiome data science using QIIME 2. *Nat Biotechnol* 2019; **37**: 852–857.

3. Callahan BJ, McMurdie PJ, Rosen MJ, Han AW, Johnson AJA, Holmes SP. DADA2: High-resolution sample inference from Illumina amplicon data. *Nat Methods* 2016; **13**: 581–583.

4. Kaehler BD, Bokulich NA, McDonald D, Knight R, Caporaso JG, Huttley GA. Species abundance information improves sequence taxonomy classification accuracy. *Nat Commun* 2019; **10**: 1–10.

5. Pruesse E, Quast C, Knittel K, Fuchs BM, Ludwig W, Peplies J, et al. SILVA: a comprehensive online resource for quality checked and aligned ribosomal RNA sequence data compatible with ARB. *Nucleic Acids Res* 2007; **35**: 7188–7196.

6. Hyatt D, Chen G-L, LoCascio PF, Land ML, Larimer FW, Hauser LJ. Prodigal: prokaryotic gene recognition and translation initiation site identification. *BMC Bioinformatics* 2010; **11**: 1–11.

7. Wu D, Jospin G, Eisen JA. Systematic identification of gene families for use as “markers” for phylogenetic and phylogeny-driven ecological studies of bacteria and archaea and their major subgroups. *PLoS One* 2013; **8**: e77033.

8. Chase AB, Karaoz U, Brodie EL, Gomez-Lunar Z, Martiny AC, Martiny JBH. Microdiversity of an abundant terrestrial bacterium encompasses extensive variation in ecologically relevant traits. *mBio* 2017; **8**: e01809-17.

9. Klau LJ, Podell S, Creamer KE, Demko AM, Singh HW, Allen EE, et al. The Natural Product Domain Seeker version 2 (NaPDoS2) webtool relates ketosynthase phylogeny to biosynthetic function. *Journal of Biological Chemistry* 2022; **298**.

10. Kent WJ. BLAT—the BLAST-like alignment tool. *Genome Res* 2002; **12**: 656–664.

11. Johnson LS, Eddy SR, Portugaly E. Hidden Markov model speed heuristic and iterative HMM search procedure. *BMC Bioinformatics* 2010; **11**: 1–8.

12. Sievers F, Wilm A, Dineen D, Gibson TJ, Karplus K, Li W, et al. Fast, scalable generation of high‐quality protein multiple sequence alignments using Clustal Omega. *Mol Syst Biol* 2011; **7**: 539.

13. Matsen FA, Kodner RB, Armbrust E. pplacer: linear time maximum-likelihood and Bayesian phylogenetic placement of sequences onto a fixed reference tree. *BMC Bioinformatics* 2010; **11**: 1–16.

14. Fu L, Niu B, Zhu Z, Wu S, Li W. CD-HIT: accelerated for clustering the next-generation sequencing data. *Bioinformatics* 2012; **28**: 3150–3152.

15. Ziemert N, Lechner A, Wietz M, Millán-Aguiñaga N, Chavarria KL, Jensen PR. Diversity and evolution of secondary metabolism in the marine actinomycete genus Salinispora. *Proceedings of the National Academy of Sciences* 2014; **111**: E1130–E1139.

16. Finn RD, Bateman A, Clements J, Coggill P, Eberhardt RY, Eddy SR, et al. Pfam: the protein families database. *Nucleic Acids Res* 2014; **42**: D222–D230.

17. Peng Y, Leung HCM, Yiu S-M, Chin FYL. IDBA-UD: a de novo assembler for single-cell and metagenomic sequencing data with highly uneven depth. *Bioinformatics* 2012; **28**: 1420–1428.

18. Blin K, Shaw S, Steinke K, Villebro R, Ziemert N, Lee SY, et al. antiSMASH 5.0: updates to the secondary metabolite genome mining pipeline. *Nucleic Acids Res* 2019; **47**: W81–W87.

19. Kautsar SA, Blin K, Shaw S, Navarro-Muñoz JC, Terlouw BR, van der Hooft JJJ, et al. MIBiG 2.0: a repository for biosynthetic gene clusters of known function. *Nucleic Acids Res* 2020; **48**: D454–D458.

20. Navarro-Muñoz JC, Selem-Mojica N, Mullowney MW, Kautsar SA, Tryon JH, Parkinson EI, et al. A computational framework to explore large-scale biosynthetic diversity. *Nat Chem Biol* 2020; **16**: 60–68.

21. Shannon P, Markiel A, Ozier O, Baliga NS, Wang JT, Ramage D, et al. Cytoscape: a software environment for integrated models of biomolecular interaction networks. *Genome Res* 2003; **13**: 2498–2504.

22. Gilchrist CLM, Chooi YH. clinker & clustermap.js: automatic generation of gene cluster comparison figures. *Bioinformatics* 2021; **37**: 2473–2475.

23. Langmead B, Salzberg SL. Fast gapped-read alignment with Bowtie 2. *Nat Methods* 2012; **9**: 357–359.

24. Kang DD, Li F, Kirton E, Thomas A, Egan R, An H, et al. MetaBAT 2: an adaptive binning algorithm for robust and efficient genome reconstruction from metagenome assemblies. *PeerJ* 2019; **7**: e7359.

25. Parks DH, Imelfort M, Skennerton CT, Hugenholtz P, Tyson GW. CheckM: assessing the quality of microbial genomes recovered from isolates, single cells, and metagenomes. *Genome Res* 2015; **25**: 1043–1055.

26. Olm MR, Brown CT, Brooks B, Banfield JF. dRep: a tool for fast and accurate genomic comparisons that enables improved genome recovery from metagenomes through de-replication. *ISME J* 2017; **11**: 2864–2868.

27. Parks DH, Chuvochina M, Rinke C, Mussig AJ, Chaumeil P-A, Hugenholtz P. GTDB: an ongoing census of bacterial and archaeal diversity through a phylogenetically consistent, rank normalized and complete genome-based taxonomy. *Nucleic Acids Res* 2022; **50**: D785–D794.

28. Chaumeil P-A, Mussig AJ, Hugenholtz P, Parks DH. GTDB-Tk: a toolkit to classify genomes with the Genome Taxonomy Database. *Bioinformatics* . 2020. Oxford University Press. , **36**: 1925–1927

29. Shaffer M, Borton MA, McGivern BB, Zayed AA, la Rosa SL 0003 3527 8101, Solden LM, et al. DRAM for distilling microbial metabolism to automate the curation of microbiome function. *Nucleic Acids Res* 2020; **48**: 8883–8900.

30. Liu J, Lopez N, Ahn Y, Goldberg T, Bromberg Y, Kerkhof LJ, et al. Novel reductive dehalogenases from the marine sponge associated bacterium Desulfoluna spongiiphila. *Environ Microbiol Rep* 2017; **9**: 537–549.

31. Nayfach S, Roux S, Seshadri R, Udwary D, Varghese N, Schulz F, et al. A genomic catalog of Earth’s microbiomes. *Nat Biotechnol* 2020; **39**: 499–509.

32. Crits-Christoph A, Diamond S, Butterfield CN, Thomas BC, Banfield JF. Novel soil bacteria possess diverse genes for secondary metabolite biosynthesis. *Nature* 2018; **558**: 440–444.

33. Stamatakis A. RAxML version 8: a tool for phylogenetic analysis and post-analysis of large phylogenies. *Bioinformatics* 2014; **30**: 1312–1313.

34. Letunic I, Bork P. Interactive Tree Of Life (iTOL) v5: an online tool for phylogenetic tree display and annotation. *Nucleic Acids Res* 2021; **49**: W293–W296.

35. Pluskal T, Castillo S, Villar-Briones A, Orešič M. MZmine 2: Modular framework for processing, visualizing, and analyzing mass spectrometry-based molecular profile data. *BMC Bioinformatics* 2010; **11**: 1–11.

36. Chong J, Soufan O, Li C, Caraus I, Li S, Bourque G, et al. MetaboAnalyst 4.0: towards more transparent and integrative metabolomics analysis. *Nucleic Acids Res* 2018; **46**: W486–W494.
