# Supplementary figures and images for: Biogeographic patterns of biosynthetic potential and specialized metabolites in marine sediments
Source: ISME J. 2023 Apr 15;17(7):976–83. doi: 10.1038/s41396-023-01410-3 (PMC10284892; doi:10.1038/s41396-023-01410-3)

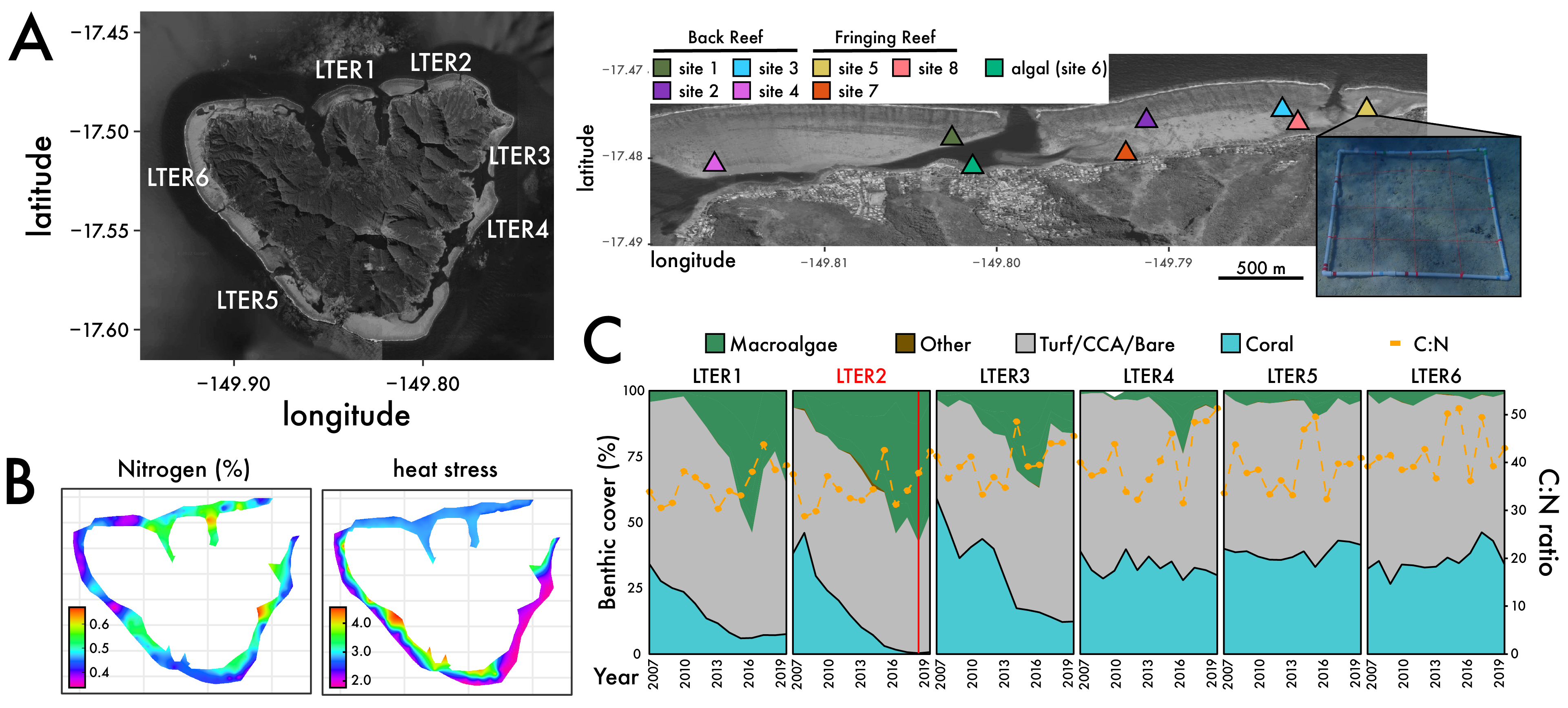

Supplement: Supplementary file 2 — Figure S1 [file 41396_2023_1410_MOESM2_ESM.png]

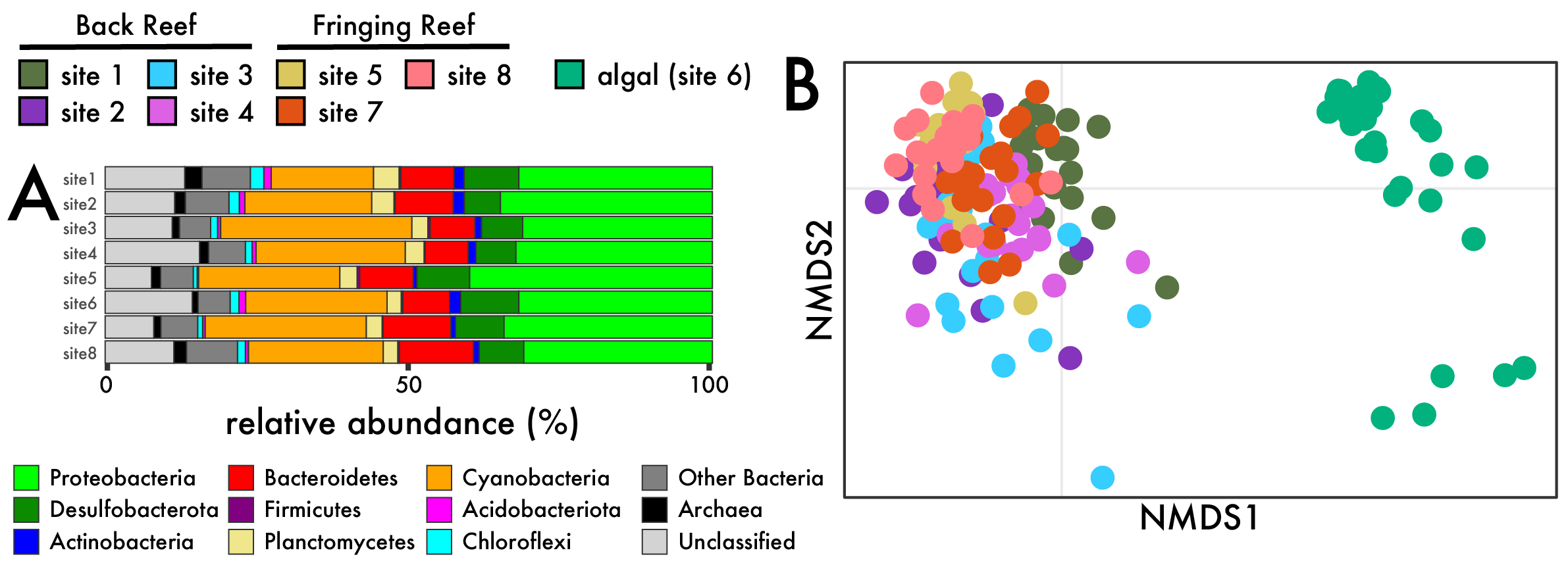

Supplement: Supplementary file 3 — Figure S2 [file 41396_2023_1410_MOESM3_ESM.png]

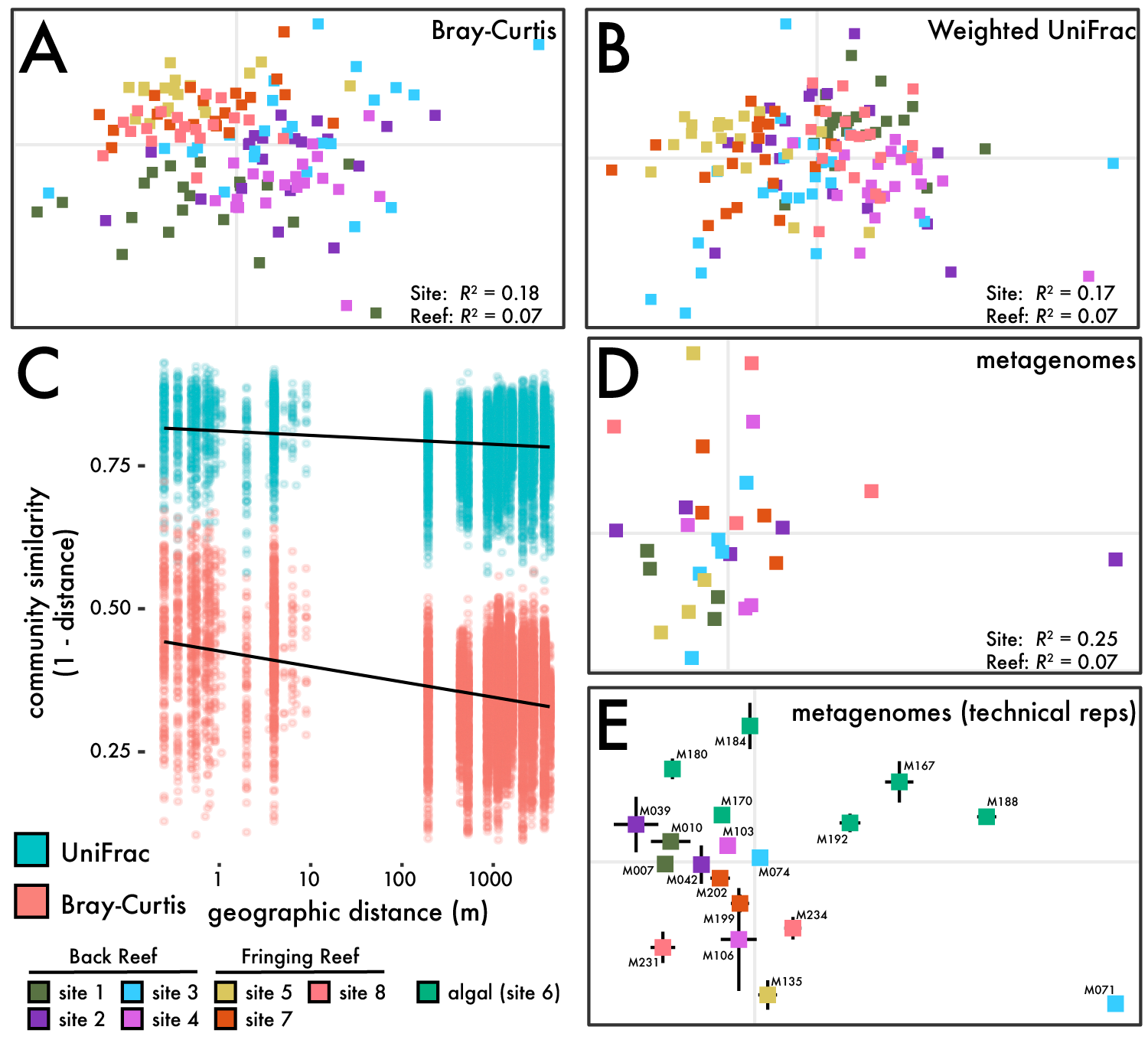

Supplement: Supplementary file 4 — Figure S3 [file 41396_2023_1410_MOESM4_ESM.png]

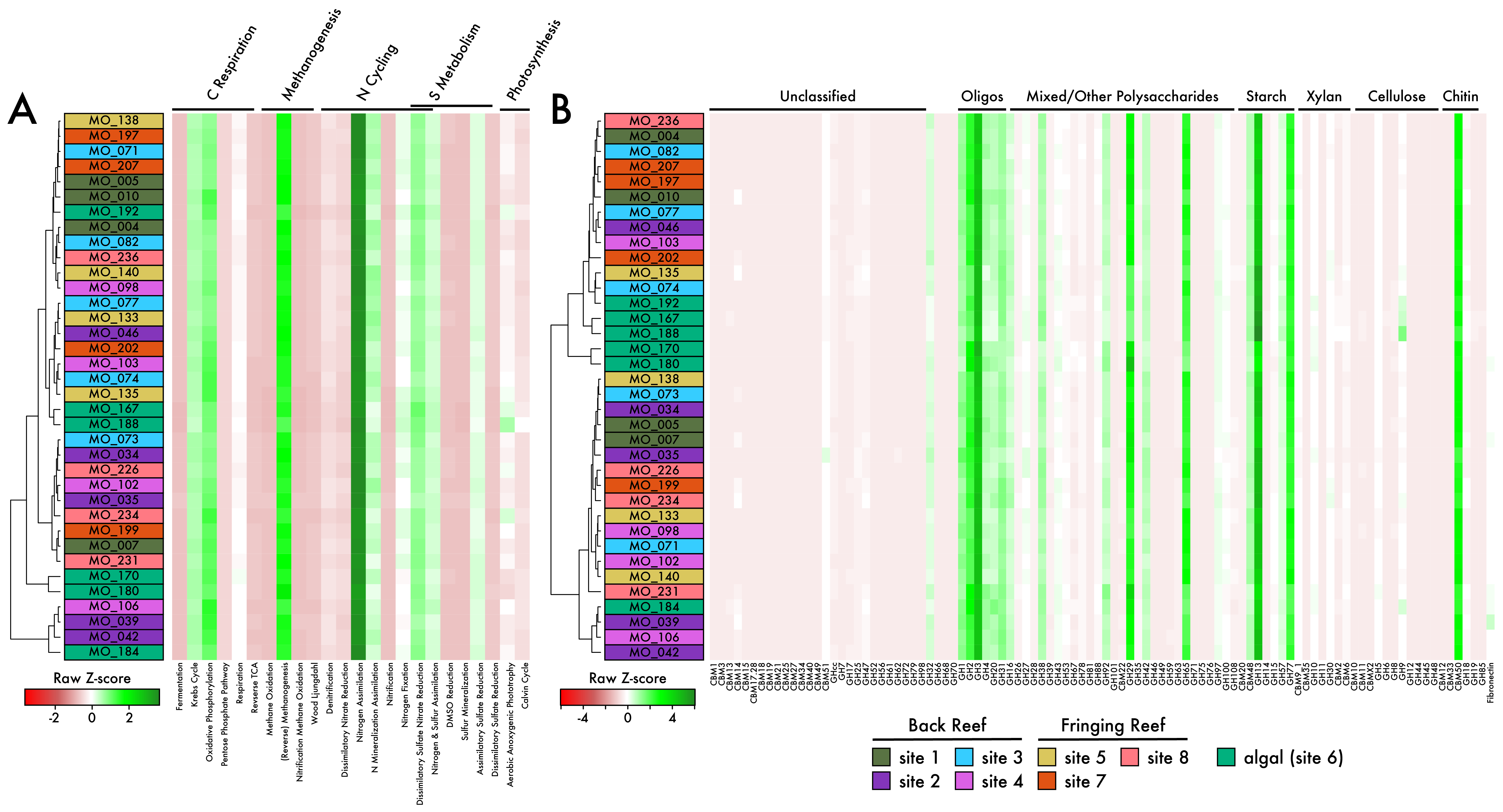

Supplement: Supplementary file 5 — Figure S4 [file 41396_2023_1410_MOESM5_ESM.png]

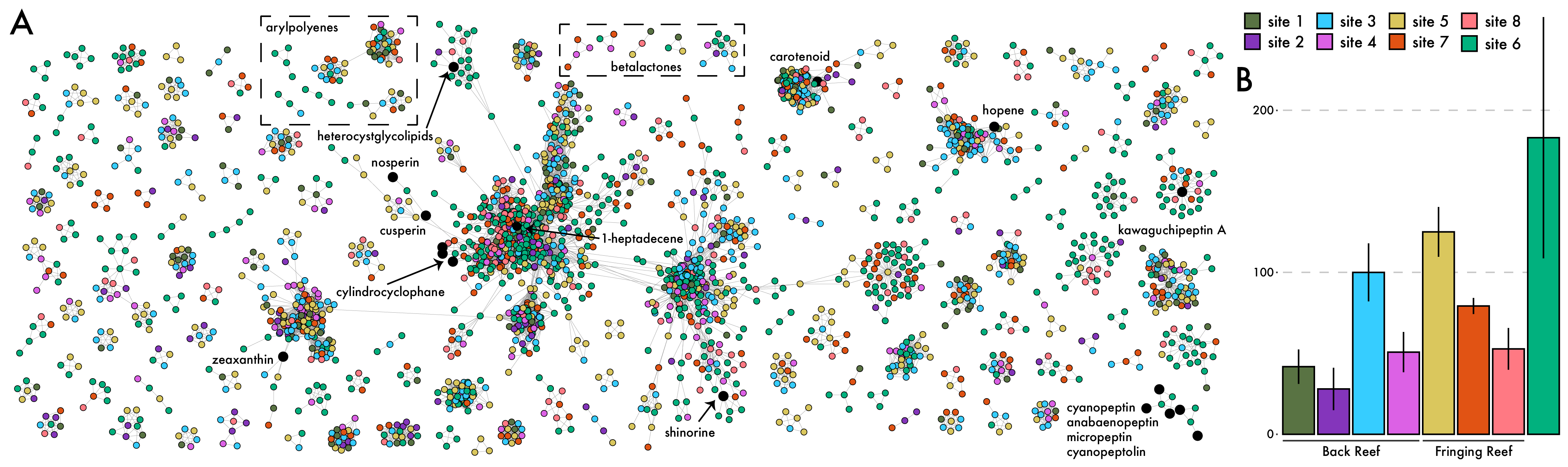

Supplement: Supplementary file 6 — Figure S5 [file 41396_2023_1410_MOESM6_ESM.png]

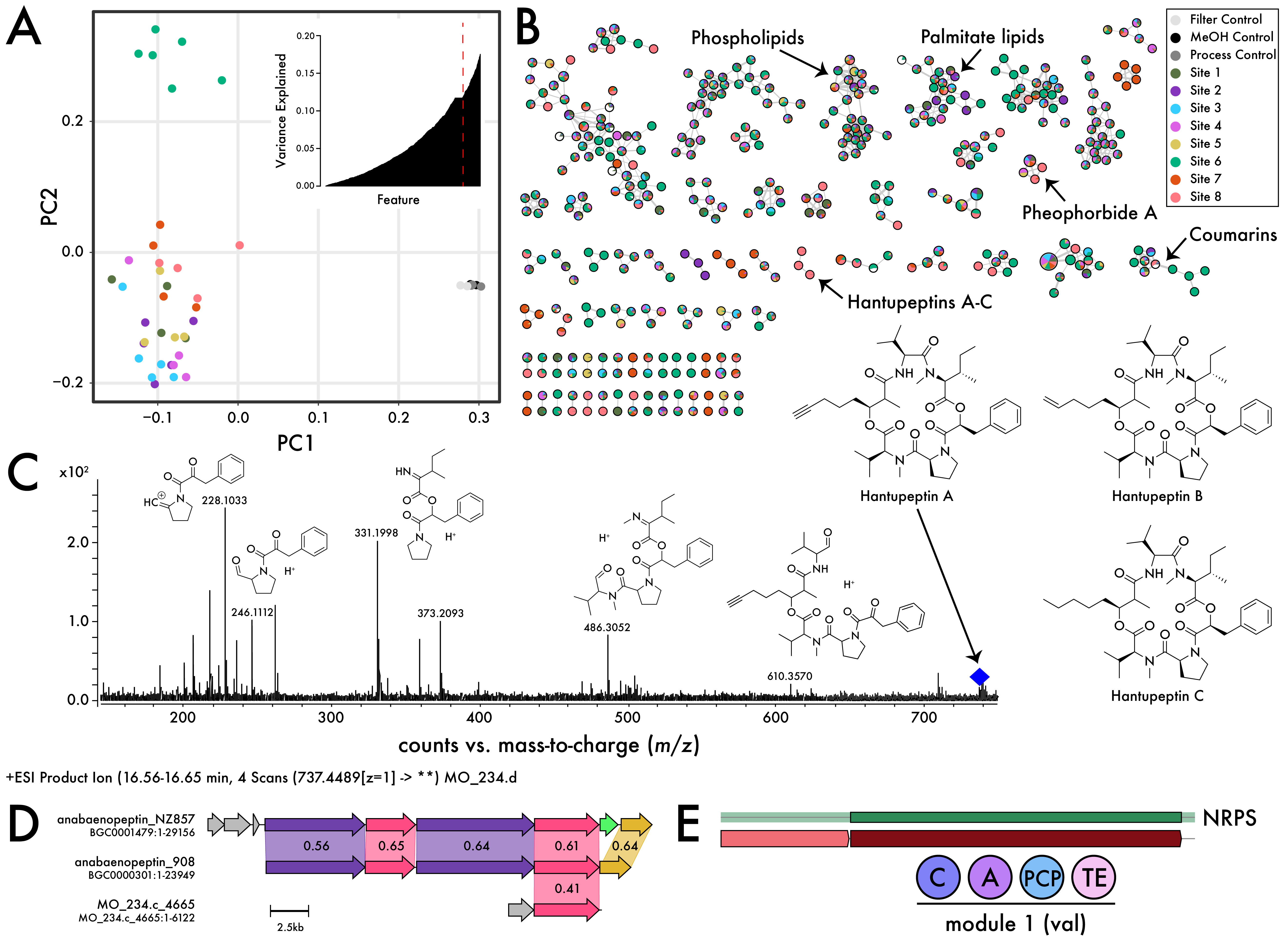

Supplement: Supplementary file 7 — Figure S6 [file 41396_2023_1410_MOESM7_ESM.png]

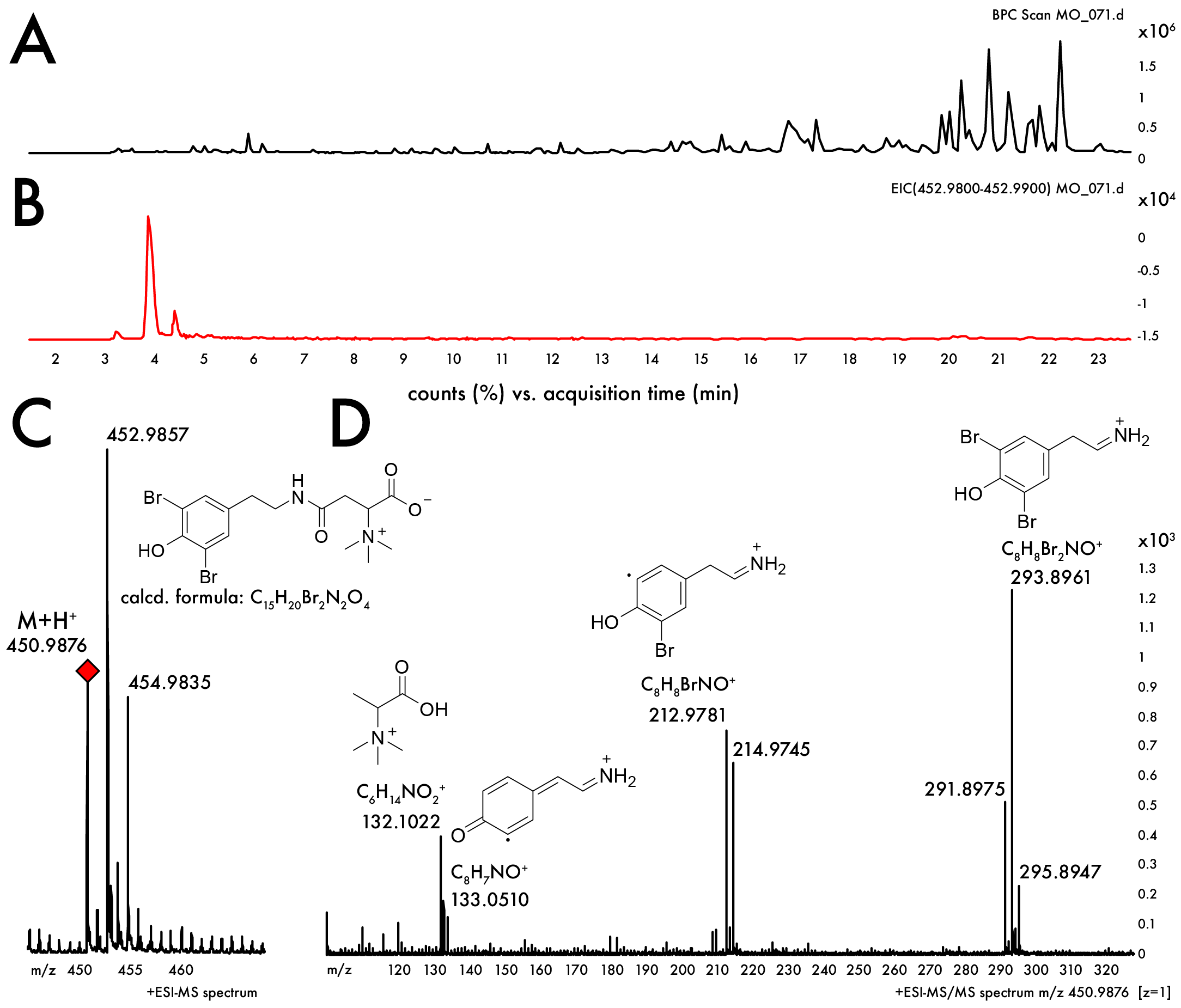

Supplement: Supplementary file 8 — Figure S7 [file 41396_2023_1410_MOESM8_ESM.png]

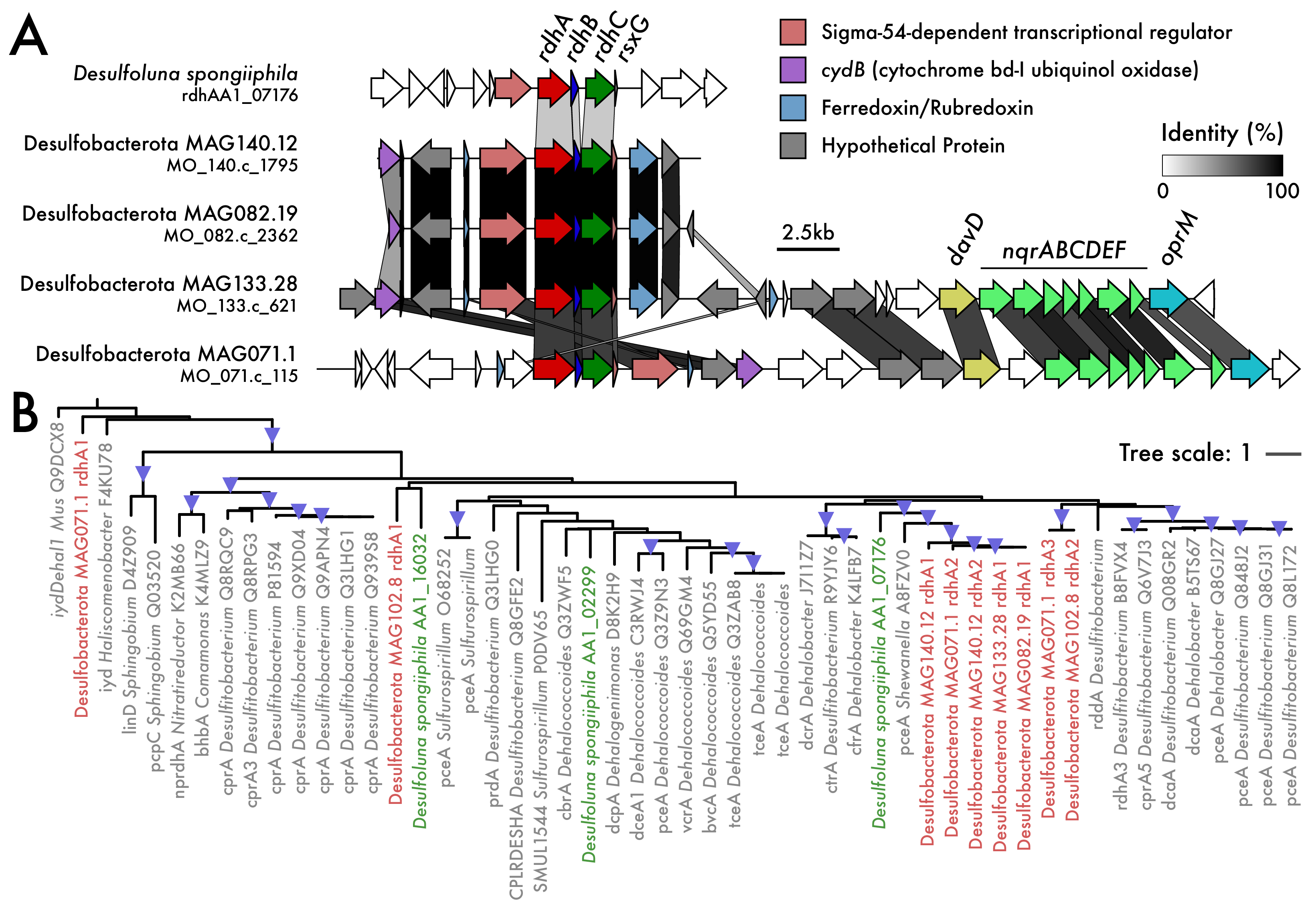

Supplement: Supplementary file 9 — Figure S8 [file 41396_2023_1410_MOESM9_ESM.png]
